# A New Prenylated Naphthoquinoid from the Aerial Parts of *Clinopodium chinense* (Benth.) O. Kuntze

**DOI:** 10.3390/molecules171213910

**Published:** 2012-11-23

**Authors:** Mingliang Zhong, Guibo Sun, Xiaopo Zhang, Guangli Sun, Xudong Xu, Shichun Yu

**Affiliations:** 1 Key Laboratory of Bioactive Substances and Resources Utilization of Chinese Herbal Medicine, Ministry of Education, Institute of Medicinal Plant Development, Chinese Academy of Medical Sciences & Peking Union Medical College, Beijing 100193, China; Email: mingliangzhongsky@163.com (M.Z.); gbsun@implad.ac.cn (G.S.); xiaopozhang2011@126.com (X.Z.); guanglisun@126.com (G.S.); 2 Department of Pharmacy, School of Pharmacy, Hebei United University, Tangshan 063000, China; 3 Beijing Hong Tai Chi Chung Medical Technology Co., Ltd., Beijing 102600, China; Email: shichunyu@hotmail.com

**Keywords:** *Clinopodium chinense* (Benth.) O. Kuntze, prenylated naphthoquinoid, (3*R*,4a*R*,10b*R*)-3,10-dihydroxy-2,2-dimethyl-3,4,4a,10b-tetrahydro-2*H*-naphtho[1,2-b]-pyran-5*H*-6-one, CD

## Abstract

A new prenylated naphthoquinoid, named (3*R*,4a*R*,10b*R*)-3,10-dihydroxy-2,2-dimethyl-3,4,4a,10b-tetrahydro-2*H*-naphtho[1,2-b]-pyran-5*H*-6-one (**1**), was isolated from the aerial parts of *Clinopodium chinense* (Benth.) O. Kuntze, together with six known compounds: apigenin (**2**), luteolin (**3**), neoeriocitrin (**4**), naringenin (**5**), narirutin (**6**), and didymin (**7**). Neoeriocitrin was isolated for the first time from the species *C. chinense*. Their structures were elucidated by spectroscopic methods, including 1D, 2D (^1^H-^1^H-COSY, HSQC, HMBC and NOESY) NMR, HR-ESI-MS. The absolute configuration of **1** was determinated using the CD method. We highlight that the structure of **1** is characterized by a rarely seen prenylated naphthoquinoid framework.

## 1. Introduction

*Clinopodium chinense* (Benth.) O. Kuntze belongs to the family Labiatae and is a perennial herb distributed in most parts of China. Its aerial parts were used as a folk medicine in China for treatment of influenza, heliosis, allergic dermatitis, dysentery, hematuria, trauma, *etc.* [1]. Modern pharmacological studies have verified that its aqueous or ethanol extracts exhibited hemostatic, anti-inflammatory, antioxidant, antibiotic activities. In addition, anti-hyperglycemic activity and protective effects in the cardiovascular system have also been reported [2]. Previous phytochemical investigations of this plant indicated the presence of flavonoids, triterpenoid saponins, and volatile oil [3,4]. In our research for bioactive constituents from this medicinal plant, a new prenylated naphthoquinoid, named (3*R*,4a*R*,10b*R*)-3,10-dihydroxy-2,2-dimethyl-3,4,4a,10b-tetrahydro-2*H*-naphtho[1,2-b]-pyran-5*H*-6-one (**1**), as well as six known flavonoids (compounds **2**–**7**) were obtained. Herein, we present the isolation and structure elucidation of compounds **1**–**7**, including the determination of absolute configuration of compound **1**by the circular dichroism method, which will provide a standard for absolute configuration determination of structures belonging to this class.

## 2. Results and Discussion

**Figure 1:**
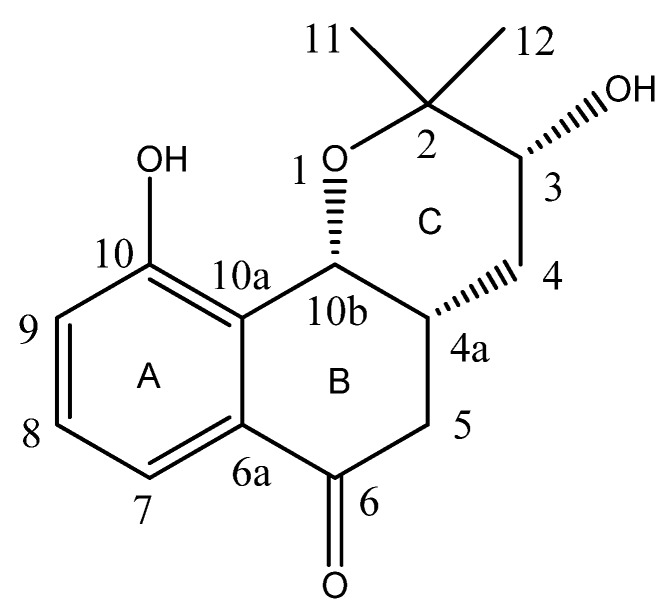
Structure of compound **1**.

**Figure 2:**
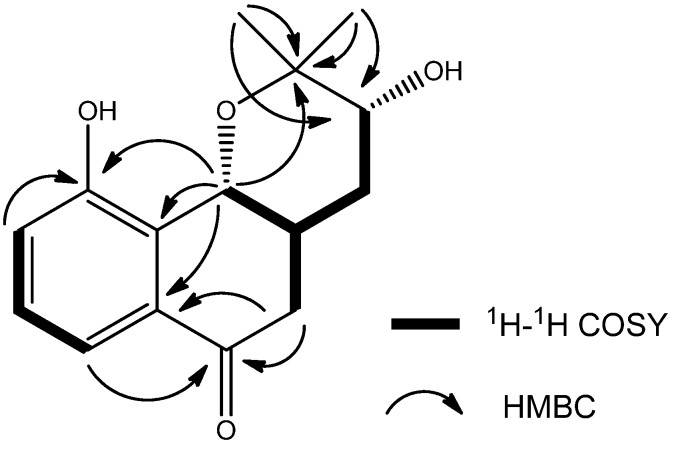
Selected HMBC (^2^J and ^3^J) correlations (**H→C**)and ^1^H-^1^H-COSY correlations of compound **1**.

Compound **1** (Figure 1) was obtained in form of colorless crystals with m.p. 151–153 °C. Its molecular formula was determined as C_15_H_18_O_4_ based on the result of HR-ESI-MS with a *quasi* molecular ion peak of [M+Na]^+^ at *m/z* 285.1098 (calcd. 285.1103). The IR spectrum of **1** showed absorptions of hydroxyl (3446 cm^−1^) and conjugated carbonyl (1680 cm^−1^) moieties [5]. The ^1^H-NMR spectrum of **1** revealed the presence of three *ortho*-coupled aromatic protons at *δ*_H_ 7.41 (1H, d, *J* = 7.8 Hz, H-7), 7.29 (1H, ps t, *J* = 7.8 Hz, H-8), 7.11 (1H, d, *J* = 7.8 Hz, H-9) and aliphatic protons at *δ*_H_ 5.14 (1H, d, *J* = 5.4 Hz, H-10b), 3.99 (1H, t, *J* = 7.8 Hz, H-3), 2.94 (1H, m, H-4a), 2.86 (1H, dd, *J* = 15.6, 9.6 Hz, H-5β), 2.59 (1H, dd, *J* = 15.6, 5.4 Hz, H-5α), 2.24 (1H, dt, *J* = 12.6, 7.8 Hz, H-4β), 1.63 (1H, ddd, *J* = 12.6, 7.8, 5.4 Hz, H-4α), 1.17 (3H, s, 11-CH_3_), 1.09 (3H, s, 12-CH_3_). The ^13^C-NMR spectrum of **1** exhibited 15 carbon signals, which were resolved through a APT experiment into two methyl, two methylene, six methine, and five quaternary carbons, including three oxygen-bearing sp^3^ carbons at *δ*_C_ 88.2 (C-3), 74.3 (C-10b) and 72.1(C-2). ^1^H-^1^H COSY correlations were observed between H-8 and H-7, H-9; H-4a and H-10b, H-4, H-5; H-4 and H-3, respectively. These correlations established the existence of two isolated proton spin-systems (Figure 2). HMBC correlations from 11-Me and 12-Me to C-2, C-3 indicated that 11-Me, 12-Me and C-3 were all connected with C-2, whereas correlations from H-10b to C-10, C-10a, C-6a and C-2 suggested that C-10b was connected with C-10a, and C-2 was linked to C-10b through a oxygen atom. The cross-peaks of H-5 and H-7 with C-6, H-5 with C-6a demonstrated that C-5 was connected with C-6, and C-6 with C-6a. The correlations from H-9 and H-10b to C-10 verified that the hydroxyl group was linked to C-10 (Figure 2). Based on the above analysis, compound **1** was assigned as a prenylated naphthoquinoid skeleton and was similar to that of 2,2-dimethyl-3-hydroxy-3,4,4a,10b-tetrahydro-5*H*-naphtho[1,2-b]-pyran-6-one [6]. The only difference was an additional hydroxyl group at position 10 in compound **1**.

**Figure 3:**
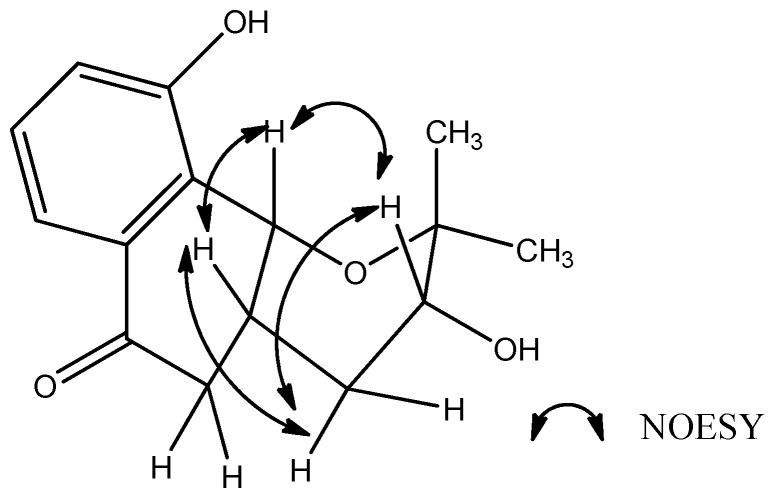
Selected NOESY correlationsof compound **1**.

The relative stereochemistry of **1** was deduced from its NOESY spectrum. The NOESY correlations between H-3 and H-10b, H-10b and H-4a (Figure 3) indicated that the junction of B/C ring adopted a *cis* configuration, suggesting H-3, H-10b, H-4a were located on the same side of the ring C.

In association with the observed NOESY correlation between H-3 and H-10b as well as the fact that this NOESY correlation could not be observed if the ring C accepted a chair form, suggesting a boat form for the ring C. The CD spectrum of **1**displayed a positive Cotton effect at 257 nm and a relatively strong negative Cotton effect at 200 nm, as well as a very weak positive Cotton effect at 343 nm, which was similar to that of *cis*-isoshinanolone, indicating the 10b*R*, 4a*R* configuration [7]. Therefore, compound **1**, a rarely seen natural prenylated naphthoquinoid, was elucidated as (3*R*,4a*R*,10b*R*)-3,10-dihydroxy-2,2-dimethyl-3,4,4a,10b-tetrahydro-2*H*-naphtho[1,2-b]-pyran-5*H*-6-one. Moreover, the established CD method regarding determination of its absolute configuration should prove useful for the structural elucidation of its analogues.

Besides the new compound, six known flavonoids, namely apigenin (**2**) [8], luteolin (**3**) [9], neoeriocitrin (**4**) [10], naringenin (**5**) [11], narirutin (**6**) [12], and didymin (**7**) [13], were isolated from the plant and their structures determinated by comparing their spectroscopic data with those reported in literature. To the best of our knowledge, neoeriocitrin was isolated for the first time from the species *C. chinense*.

## 3. Experimental

### 3.1. General

Melting points (uncorrected) were taken on a Fisher-Johns melting point apparatus. UV spectra were recorded on a Shimadzu UV-2550 UV/vis spectrophotometer. IR spectrum was acquired using a Shimadzu FTIR-8400S spectrophotometer. Optical rotations were measured by a Perkin-EImer 241 polarimeter. CD spectrum was measured on a JASCO J-815 spectrometer. The NMR data were recorded on a Bruker AV 600 instrument (600 MHz for ^1^H and 150 MHz for ^13^C) in CD_3_OD with TMS as internal standard. HR-ESI-MS data were obtained by LTQ-Orbitrap mass spectrophotometer (Thermo-Fisher Scientific, Bremen, Germany). Chromatography was performed on AB-8 macroporous resin (Chemical plant of NanKai University, Tianjin, China), silica gel (200–300 mesh, Qingdao Haiyang Chemical Factory, Qingdao, China), Sephadex LH-20 (Amersham Pharmacia Biotech AB, Uppsala, Sweden), MCI gel (75–150 μm, Mitsubishi Chemical Corporation, Tokyo, Japan) and ODS gel (40–60 μm, Daiso Co., Ltd., Osaka, Japan). Semipreparative HPLC was performed on a CXTH LC-3000 HPLC system with a CXTH LC-3000 UV spectrophotometric detector (Beijing Chuangxintongheng Science and Technology Co., Ltd., Beijing, China).

### 3.2. Plant Material

The plants were collected from Anhui Province in 2011, and identified by Dr. Jing Quan Yuan at the Key Laboratory of Bioactive Substances and Resources Utilization of Chinese Herbal Medicine, Ministry of Education, Institute of Medicinal Plant Development, Chinese Academy of Medical Sciences & Peking Union Medical College. A voucher specimen (No. 20101132) has been deposited there.

### 3.3. Extraction and Isolation

The dried aerial parts of *C. chinense* (5 kg) were decocted two times with hot water (50 L, 2 h each time), and the combined solution was concentrated under reduced pressure to yield an extract (400 g). The extract was subjected to AB-8 macroporous resin column chromatography using ethanol-H_2_O (0:100 to 95:5, v/v) and the 50%–95% fraction afforded the total flavonoids of *C. chinense* (CCF). The CCF was subjected to column chromatography on silica gel with CHCl_3_-MeOH (20:1 to 0:1, v/v), and fractionated into eight fractions (Fr.1→8). Fr.1 was subjected to ODS column chromatography eluting with CH_3_OH-H_2_O (3:7 to 1:0, v/v) to yield three subfractions (subFr.1→3). SubFr.3 was purified by Sephadex LH-20 column chromatography eluting with CH_3_OH and compound **1** was obtained after semi-preparative HPLC (15 mg). Fr.2 and Fr.3 were subjected to Sephadex LH-20 column chromatography eluting with CH_3_OH to give B1-B20 and C1-C12, respectively. B14-B16 were further separated by semi-preparative HPLC to yield compounds **2** (26 mg) and **5** (23 mg). C9 was subjected to semi-preparative HPLC to afford compound **3** (12 mg). Fr.5 was subjected to MCI column chromatography eluting with CH_3_OH-H_2_O (4:6 to 1:0, v/v) to yield 4 subfractions (subFr.1→4). SubFr.1 was applied to a semi-preparative HPLC to yield compounds **4** (12 mg) and **6** (8 mg). Compound **7** (50 mg) was crystallized in the bottle when eluted with the solvent CHCl_3_-MeOH (15:85, v/v, Fr. 4) on silica gel and then purified with MeOH.

### 3.4. Spectral Data

*(3R,4aR,10bR)-3,10-Dihydroxy-2,2-dimethyl-3,4,4a,10b-tetrahydro-2H-naphtho[1,2-b]-pyran-5H-6-one* (**1**). Colorless crystals; [α]^25^_D_: −75.0 (*c* 0.12, MeOH); UV (MeOH) *λ*_max_ (log*ε*): 205 (2.412), 220 (2.070), 257 (0.705), 315 (0.342); IR (KBr) *ν*_max_ (cm^−1^) : 3446, 1680, 1602, 1588, 1370; CD (0.076 mmol/L, MeOH): **Δ**ε_343_, +1.3, **Δ**ε_257_, +6.6, **Δ**ε_200_, −86.5; HRESI-MS *m/z*: [M+Na]^+^ 285.1098 (C_15_H_18_O_4_Na, calcd. 285.1103). For ^1^H-NMR and ^13^C-NMR (CD_3_OD) spectral data, see Table 1.

**Table 1:** ^1^H-NMR (600 MHz, CD_3_OD) and ^13^C-NMR (150 MHz, CD_3_OD) spectral data for **1**.

Position	^1^H(δ)	^13^C(δ)	HMBC
2		72.1	
3	3.99 (1H, dd, *J* = 7.8 Hz)	88.2	26.6(C-2-Me)
4	1.63 (1H, ddd, *J* = 12.6, 7.8, 5.4 Hz, 4α)	34.2	38.6(C-4a), 41.6(C-5), 74.3(C-10b), 88.2(C-3)
	2.24 (1H, dt, *J* = 12.6, 7.8 Hz, 4β)		38.6(C-4a), 72.1(C-2), 74.3(C-10b), 88.2(C-3)
4a	2.94 (1H, m)	38.6	88.2(C-3)
5	2.59 (1H, dd, *J* = 15.6, 5.4 Hz, 5α)	41.6	200.6(C-6), 34.2(C-4), 38.6(C-4a), 74.3(C-10b)
	2.86 (1H, dd, *J* = 15.6, 9.6 Hz, 5β)		134.4(C-6a), 200.6(C-6), 34.2(C-4), 38.6(C-4a), 74.3(C-10b)
6		200.6	
6a		134.4	
7	7.41 (1H, d, *J* = 7.8 Hz)	118.4	128.1(C-10a), 130.7(C-8), 200.6(C-6)
8	7.29 (1H, ps t, *J* = 7.8 Hz)	130.7	118.4(C-7), 122.4(C-9), 128.1(C-10a)
9	7.11 (1H, d, *J* = 7.8 Hz)	122.4	128.1(C-10a), 158.5(C-10)
10		158.5	
10a		128.1	
10b	5.14 (1H, d, *J* = 5.4 Hz)	74.3	128.1(C-10a), 134.4(C-6a), 158.5(C-10), 41.6(C-5), 34.2(C-4)
11-Me	1.17 (3H, s)	26.6	72.1(C-2), 88.2(C-3)
12-Me	1.09 (3H, s)	25.7	72.1(C-2), 88.2(C-3)

## 4. Conclusions

A new prenylated naphthoquinoid, named (3*R*,4a*R*,10b*R*)-3,10-dihydroxy-2,2-dimethyl-3,4,4a,10b-tetrahydro-2*H*-naphtho[1,2-b]-pyran-5*H*-6-one (**1**), was isolated from the aerial parts of *Clinopodium chinense* (Benth.) O. Kuntze. To the best of our knowledge, there were only five natural sources of this skeleton in *Lippia sidoides* [5], and *Oroxylum indicum* Vent [6], respectively. The rarely seen prenylated naphthoquinoid framework represents a new addition to the molecular diversity of *C. chinense*. Additionally, the deduced absolute configuration of **1** by the circular dichroism method will provide an approach applicable to determining the stereochemistry of other prenylated naphthoquinoid derivatives.

## References

[1] Chi H.D., Lu J.C. (2007). Advances in studies on medicinal plants in *Clinopodium*. J. Shenyang Pharm. Univ..

[2] Zhong M.L., Xu X.D., Yu S.C., Sun G.L. (2012). Advances in studies on medicinal plants in Clinopodium Linn. Chin. Tradit. Herbal Drugs.

[3] Miyase T., Matsushima Y. (1997). Saikosaponin homologs from *Clinopodium* spp. The structures of clinoposaponins XII–XX. Chem. Pharm. Bull..

[4] Liu Z.M., Jia Z.J., Gates R.G., Li D., Owen N. (1995). Triterpenoid saponins from *Clinopodium.chinensis*. J. Nat. Prod..

[5] Macambira L.M.A., Andrade C.H.S., Matos F.J.A., Craveiro A.A., Braz Filho R. (1986). Naphthoquinoids from *Lippia Sidoids*. J. Nat. Prod..

[6] Kizu H., Habe S., Ishida M., Tomimori T. (1994). Studies on the nepalese crude drugs. XVII. On the naphthalene related compounds from the root bark of *Oroxylum indicum* Vent. Yakugaku Zasshi.

[7] Bringmann G., Messer K., Saeb W., Peters E.M., Peters K. (2001). The absolute configuration of (+)-isoshinaolone and in situ LC-CD analysis of its stereoisomer from crude extracts. Phytochemistry.

[8] Fathiazad F., Ddlazar A., Amiri R., Sarker S. (2006). Extraction of flavonoids and quantification of rutin from waste tobacco leaves. Iran. J. Pharm. Res..

[9] Owen R.W., Haubner R., Mier W., Giacosa A., Hull W.E., Spiegelhalder B., Bartsch H. (2003). Isolation, structure elucidation and antioxidant potential of the major phenolic and flavonoid compounds in brined olive drupes. Food Chem. Toxicol..

[10] Li F., Meng F., Xiong Z., Li Y., Liu R., Liu H. (2006). Stimulative activity of *Drynaria fortunei* (Kunze) J. Sm. extracts and two of its flavonoids on the proliferation of osteoblastic like cells. Pharmazie.

[11] Yamauchi K., Mitsunaga T., Batubara I. (2011). Isolation, identification and tyrosinase inhibitory avtivities of the extractives from *Allamanda cathartica*. Nat. Resour..

[12] Shimoda K., Kubota N., Taniuchi K., Sato D., Nakajima N., Hamada H., Hamada H. (2010). Biotransformation of naringin and naringenin by cultured *Eucalyptus perriniana* cells. Phytochemistry.

[13] Liu R.M., Kong L.Y., Li A.F., Sun A.L. (2007). Preparative Isolation and Purification of Saponin and Flavone Glycoside Compounds from *Clinopodium chinense* (Benth) O. Kuntze by High Speed Countercurrent Chromatography. J. Liq. Chromatogr. Relat. Technol..

